# Deoxyhaemoglobin as a biomarker of cerebral autoregulation

**DOI:** 10.1186/cc10902

**Published:** 2012-03-20

**Authors:** D Highton, A Ghosh, I Tachtsidis, C Kolyva, J Panovska, C Elwell, M Smith

**Affiliations:** 1 National Hospital for Neurology and Neurosurgery, London, UK; 2 UCL, London, UK

## Introduction

Cerebral autoregulation (CA) maintains cerebral blood flow over a range of perfusion pressure. Continuous CA monitoring might define pressure targets minimising secondary brain injury, but application is limited by available monitoring modalities. Near-infrared spectroscopy (NIRS) is a noninvasive optical technique characterising aspects of CA. The NIRS-derived tissue oxygenation index (TOI) is correlated with blood pressure (BP) to produce an index of vascular reactivity (TOx) [1]. The contribution from extracerebral tissues, optical complexity of injured brain and complex physiology represented by NIRS are likely to limit agreement with other techniques. NIRS-measured deoxyhaemoglobin (HHb) may have advantages as its physiological confounds are less complicated and are predominantly in the cerebral venous circulation. This study compares HHb with established indices of reactivity - the mean velocity index (Mx) and oxygen reactivity index (ORx).

## Methods

Thirteen brain-injured patients were studied. Ipsilateral 60-minute recordings included intracranial pressure, brain tissue oxygen (PbrO_2_), transcranial Doppler and NIRS (NIRO 100; Hamamatsu Photonics). ORx and Mx were derived from continuous correlation between BP and neuromonitoring [1]. HHb was compared identically deriving HHBx. Comparisons used Pearson correlation, subsequent analysis characterised time lags between BP and monitored variables (0.05 to 0.003 Hz) with wavelet lag coherence.

## Results

There was correlation between HHBx (*r *= -0.62, *P *< 0.01), ORx (*r *= 0.52, *P *< 0.05) and Mx. TOx showed no significant correlation (*r *= 0.18) as individual recordings demonstrated TOI fluctuations paradoxical to other monitoring. The mean lag between BP and HHb (24 seconds) was shorter than PbrO_2 _(68 seconds).

## Conclusion

HHb may provide a surrogate to inform cerebrovascular reactivity assessment. Complexity in the oxyhaemoglobin component of TOI may be introduced by vasopressor-related skin artefact or arterial volume changes [2] explaining poor agreement of TOx. HHb is theoretically free of this effect but will vary with cerebral metabolism, venous dynamics and oxygenation and demonstrates lag behind BP changes. Future analyses might compensate using model-based analysis [3], potentially describing measures of vascular reactivity from multiple NIRS and neuromonitoring variables, incorporating widely different aspects of cerebral physiology.
